# Phosphoinositide 3-kinase targeting by the β galactoside binding protein cytokine negates akt gene expression and leads aggressive breast cancer cells to apoptotic death

**DOI:** 10.1186/bcr2217

**Published:** 2009-01-08

**Authors:** Valerie Wells, Livio Mallucci

**Affiliations:** 1 Cell Signalling and Growth Laboratory, School of Biomedical and Health Sciences, King's College London, Franklin Wilkins Building, 150 Stamford Street, London SE1 9NH, UK

## Abstract

**Introduction:**

Phosphoinositide 3-kinase (PI3K)-activated signalling has a critical role in the evolution of aggressive tumourigenesis and is therefore a prime target for anticancer therapy. Previously we have shown that the β galactoside binding protein (βGBP) cytokine, an antiproliferative molecule, induces functional inhibition of class 1A and class 1B PI3K. Here, we have investigated whether, by targeting PI3K, βGBP has therapeutic efficacy in aggressive breast cancer cells where strong mitogenic input is fuelled by overexpression of the ErbB2 (also known as HER/neu, for human epidermal growth factor receptor 2) oncoprotein receptor and have used immortalised ductal cells and non-aggressive mammary cancer cells, which express ErbB2 at low levels, as controls.

**Methods:**

Aggressive BT474 and SKBR3 cancer cells where ErbB2 is overexpressed, MCF10A immortalised ductal cells and non-invasive MCF-7 cancer cells which express low levels of ErbB2, both in their naive state and when forced to mimic aggressive behaviour, were used. Class IA PI3K was immunoprecipitated and the conversion of phosphatidylinositol (4,5)-biphosphate (PIP2) to phosphatidylinositol (3,4,5)-trisphosphate (PIP3) assessed by ELISA. The consequences of PI3K inhibition by βGBP were analysed at proliferation level, by extracellular signal-regulated kinase (ERK) activation, by akt gene expression and by apoptosis. Apoptosis was documented by changes in mitochondrial membrane potential, alteration of the plasma membrane, caspase 3 activation and DNA fragmentation. Phosphorylated and total ERK were measured by Western blot analysis and akt mRNA levels by Northern blot analysis. The results obtained with the BT474 and SKBR3 cells were validated in the MCF10A ductal cells and in non-invasive MCF-7 breast cancer cells forced into mimicking the *in vitro *behaviour of the BT474 and SKBR3 cells.

**Results:**

In aggressive breast cancer cells, where mitogenic signalling is enforced by the ErbB2 oncoprotein receptor, functional inhibition of the catalytic activity of PI3K by the βGBP cytokine and loss of akt mRNA results in apoptotic death. A functional correlation between ERK and the kt gene was also found. The relationship between ERK, akt mRNA, PI3K and cell vulnerability to βGBP challenge was sustained both in mammary ductal cells forced to mimic an aggressive behaviour and in non-aggressive breast cancer cells undergoing an enforced shift into an aggressive phenotype.

**Conclusions:**

βGBP, a newly discovered physiological inhibitor of PI3K, is a selective and potent inducer of apoptosis in aggressive breast cancer cells. Due to its physiological nature, which carries no chemotherapeutic disadvantages, βGBP has the potential to be safely tested in clinical trials.

## Introduction

The biological behaviour of cancer cells and their response to therapies is determined by their mutational repertoire, of which change leading to enhanced mitogenic signalling is one aspect. Genetic alterations, which in cancer cells magnify mitogenic signalling and are a cause of aggressive disease and resistance to therapies, include amplification of the ErbB2 (also known as HER/neu, for human epidermal growth factor receptor 2) gene, present in many types of cancer and frequent in breast, ovarian and stomach carcinomas [1].

ErbB2 is a ligand-less member of the ErbB/epidermal growth factor (EGF) tyrosine kinase receptor family that enhances mitogenic signalling: by being constitutively active, by dimerising as a preferred partner with other ErbB members that in breast cancer can also be overexpressed, and by resisting endocytic degradation and returning to the cell surface [2-5]. Phosphorylated tyrosine residues in the cytoplasmic tail of the ErbB2 molecule lead to the formation of high affinity binding sites for the Src homology 2 (SH2) domains of Src homology 2 containing (Shc) and growth factor receptor-bound protein 2 (Grb2) adapter proteins [6,7], the binding of the nucleotide exchange factor son of Sevenless (SOS) to the SH3 domains of Grb2 and the conversion of GDP-Ras to active GTP-Ras which mediates the activation of effector pathways that transduce proliferative signalling [8,9]. Critically, by interacting with the catalytic subunits of class IA [10] and class IB [11-13] phosphoinositide 3-kinase (PI3K), activated Ras can contribute to coupling mitogenic input with survival ability.

Class I PI3Ks are a central feature of many signalling pathways that allow cells to withstand apoptotic stimuli and secure mitogenic expansion. By catalysing the conversion of phosphatidylinositol (4,5)-biphosphate (PIP2) to phosphatidylinositol (3,4,5)-trisphosphate (PIP3), PI3K enables Akt/protein Kinase B (PKB) recruitment to the plasma membrane where Akt is activated to become the principal effector of survival signalling [14,15]. Phosphorylation of downstream targets such as Bad, forkhead transcription factors, IκB kinase (IKKα), caspase 9 and Yes-associated proteins (YAPs) by activated Akt confers resistance to apoptosis [16-19]. Furthermore, activated Akt has also a role in promoting cell growth and cell proliferation via phosphorylation and repression of the forkhead box O (FOXO) family of transcription factors and phosphorylation and inhibition of glycogen synthetase kinase-3β [20].

Class IA PI3K is specifically implicated in the pathogenesis of cancer. High frequency of somatic mutations in the PI3K catalytic subunit (PI3KCA) gene [21,22], results in constitutively active mutants which have the capacity to transform normal cells into cancer cells and to be oncogenic *in vivo *[23-25]. The importance of PI3K in cancerogenesis is further indicated by the evidence that many aggressive and drug resistant tumour cells display elevated levels of PIP3 as a result of phosphatase and tensin homolog (PTEN) deletion [26].

The role of the PI3K signalling network in cell proliferation, cell survival and, through PI3K interaction with Rac proteins, in cell motility and migration [15,27], all processes of central importance to the evolution of aggressive tumourigenesis, has provided scope for the design of anticancer drugs aimed at PI3K and its downstream effectors [28,29,18]. However, there is now evidence that inhibition of PI3K activity can be achieved without chemotherapeutic disadvantages following physiological routes. We have recently shown that monomeric β-galactoside binding protein (βGBP), a molecule that we first discovered to be an endogenous negative cell cycle regulator [30] and that we then identified as a cytokine [31], is a natural physiological inhibitor of class IA and class IB PI3K [32]. Through functional inhibition of p110αβ, βGBP induces downregulation of PI3K activity, suppression of Ras-GTP loading, consequent loss of extracellular signal-regulated kinase (ERK) activation and block of cell proliferation [32].

In this study we have used the recombinant form of the human βGBP cytokine to investigate its effect in aggressive cancer cells where the ErbB2 oncoprotein receptor is overexpressed, taking as a paradigm cancer of the breast, known for high mutation frequency in the gene encoding the p110α subunit of PI3K [22,25]. In addition we have used immortalised mammary ductal cells and non-invasive breast cancer cells, where ErbB2 is at low levels, both in their naïve state and when forced to mimic aggressiveness as represented by the *in vitro *behaviour of the cells which overexpress ErbB2.

We provide the first evidence that PI3K activity is a requirement for akt gene expression and that inhibition of PI3K activity by the βGBP cytokine and loss of Akt gene expression is followed by apoptotic death in ErbB2 aggressive cancer cells and in cells forced to mimic their *in vitro *behaviour, but not in naïve mammary ductal cells.

## Materials and methods

### Cell lines

The BT474 cells (Cancer Research UK, London, UK) were cultured in DMEM/F12 with 10% foetal calf serum (FCS) (Invitrogen, Renfrew, UK) and 20 μg/ml insulin (Sigma, Dorset, UK); the SKBR3 cells (Cancer Research UK) were grown in DMEM (Invitrogen) plus 10% FCS. MCF10A, MCF10A^V12Ras ^(Cancer Research UK) and MCF10A^CTx ^cells were grown in DMEM/F12 plus 5% horse serum (Invitrogen), 10 μg/ml insulin, 5 μg/ml hydrocortisone (Calbiochem, San Diego, CA, USA) and 20 μg/ml epidermal growth factor (Calbiochem), plus 100 ng/ml cholera toxin (Sigma) in the case of the MCF10A^CTx ^cells. Cultures were incubated at 37°C in a humidified atmosphere of 5% CO_2 _in air.

### Apoptosis assays

Tetramethylrhodamine ethyl ester (TMRE) (Molecular Probes/Invitrogen) staining was used to assess loss of mitochondrial membrane potential. Redistribution of plasma membrane phosphatidylserine was assessed using annexin V-fluorescein isothiocyanate (FITC) (Pharmingen, San Diego, CA, USA). Caspase 3 activity was measured by cleavage of non-fluorescent PhiPhiLux to a fluorescent product (Oncoimmunin, Gaithersburg, MD, USA). Strand break DNA fragmentation was analysed by terminal deoxynucleotidyl transferase (TdT)-mediated dUTP nick-end labelling (TUNEL) using the Apo-Brdu kit (Phoenix Flow Systems, Phoenix, AZ, USA)) and analysed by fluorescence-activated cell sorting (FACS) using a FACS Calibur (Becton Dickinson, San Jose, CA, USA) system. All methods were carried out according to the manufacturers' instructions.

### PI3K assays

For direct functional assessment of PI3K activity, class IA PI3K was isolated by immunoprecipitation using an antibody to the p85 adapter subunit and the ability of the coprecipitated catalytic p110 catalytic subunit to convert a standard PIP2 to PIP3 in a kinase reaction assessed by measuring the generated PIP3 by competitive ELISA.

5 × 10^6 ^cells were washed three times with 137 mM NaCl, 20 mM Tris HCl pH7.4, 1 mM CaCl_2_, 1 mM MgCl_2_, 0.1 mM Na orthovanadate (Sigma) (buffer A) and lysed in 1 ml of the same buffer supplemented with 1 mM phenylmethylsulphonyl fluoride (PMSF) (Sigma) and 1% nonyl phenoxylpolyethoxylethanol (NP40) (Calbiochem) for 20 min on ice. Lysates were centrifuged at 13,000 rpm for 10 min to remove insoluble material and the supernatants stored at -80°C. Frozen lysates containing 600 μg protein were thawed on ice and PI3K was immunoprecipitated by incubation with 5 μl anti-PI3K p85 (Upstate Biotechnology, Lake Placid, NY, USA) for 1 h at 4°C on a rotating wheel, followed by addition of 60 μl of a 50% slurry of Protein A agarose (Sigma) beads in PBS for 1 h at 4°C. The immunoprecipitated enzyme was collected by centrifugation at 13,000 rpm for 10 s. Pellets were washed three times in buffer A plus 1% NP40, three times in 0.1 M Tris-HCl, pH 7.4, 5 mM LiCl, 0.1 mM Na orthovanadate and twice with 10 mM Tris-HCl, pH 7.4, 150 mM NaCl, 5 mM ethylenediaminetetraacetic acid (EDTA), 0.1 mM Na orthovanadate. Pellets resuspended in 110 μl kinase reaction buffer (5 mM 4-(2-hydroxyethyl)-1-piperazineethanesulfonic acid (HEPES) pH 7.0, 2.5 mM MgCl_2_, 25 μM ATP) were incubated in a water bath for 3 h at 37°C with 40 pmol PI(4,5)P2 substrate (Echelon Biosciences, Salt Lake City, UT, USA). The reaction was stopped with EDTA at a final concentration of 5 mM and the reaction mixture centrifuged at 13,000 rpm at 4°C. Supernatants were transferred to a microtitre plate for a competitive ELISA (Echelon Biosciences K-1000) to quantify the PIP3 generated in the kinase reaction. Duplicate 50 μl volumes of the supernatants were each incubated with 50 μl of anti-PIP3 antibody for 1 h at room temperature. The reaction mixture was then transferred to a microtitre plate coated with PIP3 and incubated for 1 h in the dark. After three washes with Tris-buffered saline (TBS) plus 0.05% Tween 20 (Sigma), 100 μl of horseradish peroxidase (HRP)-conjugated antibody to the anti-PIP3 was added to each well and incubated for 1 h at room temperature in the dark. Following three further washes with TBS plus 0.05% Tween 20, 100 μl of tetramethyl benzidine (TMB) substrate (Echelon Biosciences) was added and the reaction was stopped after an appropriate time (approximately 20 min) with 100 μl 0.5 M H_2_SO_4_. Absorbance of the samples was measured at 450 nm and the PIP3 was quantified by comparison with a PIP3 standard curve carried out in parallel with the experimental samples and plotted on a log scale.

### Northern blot analysis

Total RNA was extracted from cells using Trizol reagent (Invitrogen) according to the manufacturer's instructions. A total of 10 μg RNA was run on 2.2 M formaldehyde/1.25% agarose gels. akt mRNA was assessed using cDNA probe HA. akt, which recognises akt gene 1,2,3 (a gift from Julian Downward, Cancer Research UK, London, UK). A glyceraldehyde 3-phosphate dehydrogenase (GAPDH) cDNA probe (Cancer Research UK) was used as an RNA loading control.

### Western blot analysis

Phosphorylated ERK1/2 were probed with 1:1,000 anti-phospho-p44 ERK1 and p42 ERK2 monoclonal antibody (Cell Signaling Technology). Non-phosphorylated ERK1/2 proteins were probed with 1:1,000 anti-ERK2 (Santa Cruz Biotechnology, Santa Cruz, CA, USA), which recognises both p44 ERK1 and p42 ERK2. Phosphorylated Akt was detected using 1:1,000 anti-phospho-Akt (Ser473) antibody (Cell Signaling Technology) and total Akt1/2 protein was probed with 1:1000 anti-Akt1/2 (H136) (Santa Cruz). Secondary antibodies conjugated to HRP (GE Healthcare, Chalfont St Giles, UK) were used at 1:1,000 dilution and visualised by enhanced chemiluminescence (GE Healthcare).

### Recombinant βGBP

Human recombinant βGBP (Hu-r-βGBP) was expressed in *Escherichia coli *BL21(DE3) using hGal-1 cDNA in PET21a, purified by lactose-agarose (Sigma) affinity chromatography and purity assessed by matrix-assisted laser desorption/ionization-time of flight (MALDI-TOF) spectrometry.

### Metabolic inhibitors

The mitogen-activated protein kinase kinase (MEK) inhibitor UO126 (Calbiochem) was added to naïve MCF10A, MCF10A^CTx ^and MCF10AV12Ras cells 3 h after seeding at concentrations of 10 μM, 1 μM, 100 nM and 10 nM (IC50 for MEK1, 72 nM, MEK2, 58 nM) and cell viability, cell numbers and inhibition of ERK1/2 were assessed in parallel.

## Results

### Apoptosis: correlation between inhibition of PI3K activity and akt gene suppression

**Figure 1:**
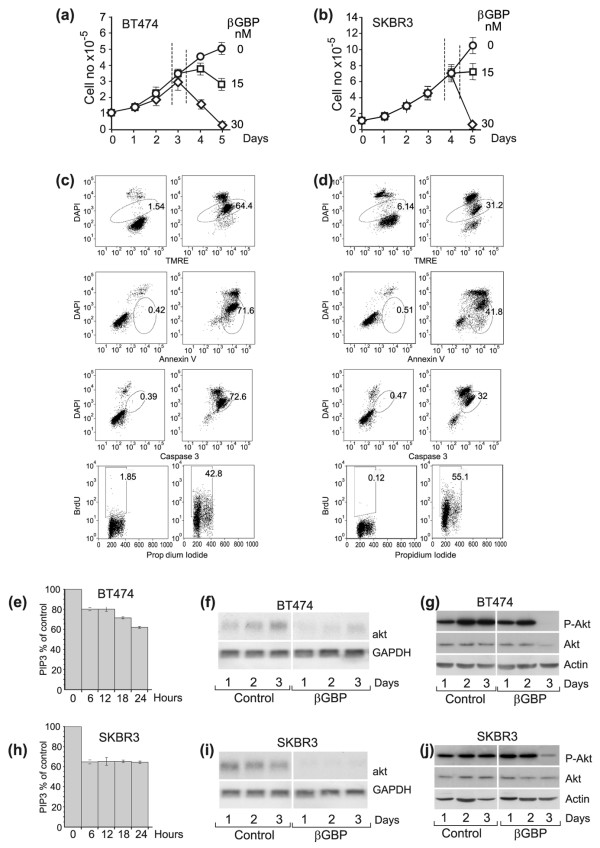
**Events leading to apoptotic death in BT474 and SKBR3 breast cancer cells treated with human recombinant β galactoside binding protein (Hu-r-βGBP)**. **(a-b)**Rate of cell proliferation and dose response to Hu-r-βGBP. Dotted lines in growth curves define the time of occurrence of apoptotic events. Values are means of triplicate cultures ± standard error of the mean (SEM). **(c-d)**Sequential expression of apoptotic events after treatment with 30 nM Hu-r-βGBP. Top to bottom: loss of mitochondrial membrane potential assessed by tetramethylrhodamine ester (TMRE); phosphatidylserine orientation at the plasma membrane assessed by annexin V staining; caspase 3 activity and DNA fragmentation (terminal deoxynucleotidyl transferase (TdT)-mediated dUTP nick-end labelling (TUNEL)). Apoptotic cells are in circled areas. Values are percentages of cells in apoptosis. Left panels: controls, right panels: cells treated with 30 nM Hu-r-βGBP. **(e, h)**Inhibition of class IA phosphoinositide 3-kinase (PI3K) activity by 30 nM Hu-r-βGBP measured as phosphatidylinositol (3,4,5)-trisphosphate (PIP3) generated from immunoprecipitated PI3K in a kinase assay at 30°C, and assessed in a competitive ELISA. Values are means from triplicate readings ± SEM. **(f, i) **akt mRNA and glyceraldehyde 3-phosphate dehydrogenase (GAPDH) mRNA levels. Left panels: controls, right panels: cells treated with 30 nM Hu-r-βGBP. **(g, j)**Phosphorylated (Ser473) Akt and total Akt protein. Left panels: controls, right panels: cells treated with 30 nM Hu-r-βGBP. **(a-j) **Hu-r-βGBP added at 3 h after seeding. The data are representative of results obtained in three separate experiments.

To determine whether βGBP could overcome the strength of endogenous mitogenic signalling in aggressive cancers we examined BT474 and SKBR3 breast cancer cells that express high levels of ErbB2 [33]. We found that βGBP had virtually no effect on cell replication (Figure 1a, b) until, after two to three generation cycles, abrupt cell death was triggered by an acute sequence of apoptotic events documented by changes in mitochondrial membrane potential as assessed by TMRE staining, by functional alteration of the plasma membrane as assessed by annexin V staining, by caspase 3 activation and by DNA fragmentation as assessed by TUNEL analysis (Figure 1c, d). We found, predictably, no changes in ERK phosphorylation while cell replication continued unaffected but found, as already observed in the normal cell context [32], that βGBP had affected PI3K function.

As cell phosphoinositide levels do not directly represent the functional state of the PI3K enzyme, but are the result of PI3K and PTEN activity, to estimate PI3K enzymatic activity we isolated class IA PI3K by immunoprecipitation using an antibody to the p85α adapter subunit and assessed the ability of the coprecipitated p110 catalytic subunit to convert a standard PIP2 to PIP3 in a kinase reaction by measuring the generated PIP3 in a competitive ELISA. Figure 1e, h demonstrates that downregulation of PI3K activity was an early event already present at 6 h after the addition of βGBP. Following inhibition of PI3K activity, we detected loss of phosphorylated Akt and loss of Akt protein preceding the apoptotic process (Figure 1g, j; right panels), though less promptly in the SKBR3 cells where cell proliferation in the presence of βGBP extended for 1 day longer (Figure 1b).

To investigate the cause for the loss of the Akt protein we assessed akt mRNA levels. Figure 1f, i shows that akt mRNA, clearly expressed in the unchallenged controls (left panels), within 1 day from the addition of βGBP, had become either undetectable (Figure 1i, right panel) or very faintly expressed (Figure 1f, right panel), a likely last effort to survive before undergoing apoptotic death. Framed within a time sequence, the above observations show that treatment with βGBP resulted in downregulation of PI3K activity, loss of akt mRNA, loss of Akt and apoptosis.

### Mitogenic input, akt mRNA levels and apoptosis

Based on the evidence shown in Figure 1, we hypothesised that to elevate mitogenic input, corresponding elevated survival signalling may create conditions that foster mitogenic expansion and cell survival, and also that akt gene expression requires PI3K activity, and that by downregulation of PI3K activity and consequent suppression of akt gene function, βGBP triggers apoptosis. To test the validity of this assumption we experimentally enforced mitogenic pressure in non-cancerous cells. We used spontaneously immortalised MCF10A mammary ductal cells which have recognisable normal appearance and behaviour [34], MCF10A cells where mitogenic input was enhanced by the addition of cholera toxin [MCF10A^CTx^] which increases ERK activity via adenyl cyclase upregulation [35], and MCFI0A cells stably transfected with constitutively active p21 Ras mutated at valine 12 (MCF10A^V12Ras^), which strongly activates Raf-ERK signalling [36].

**Figure 2:**
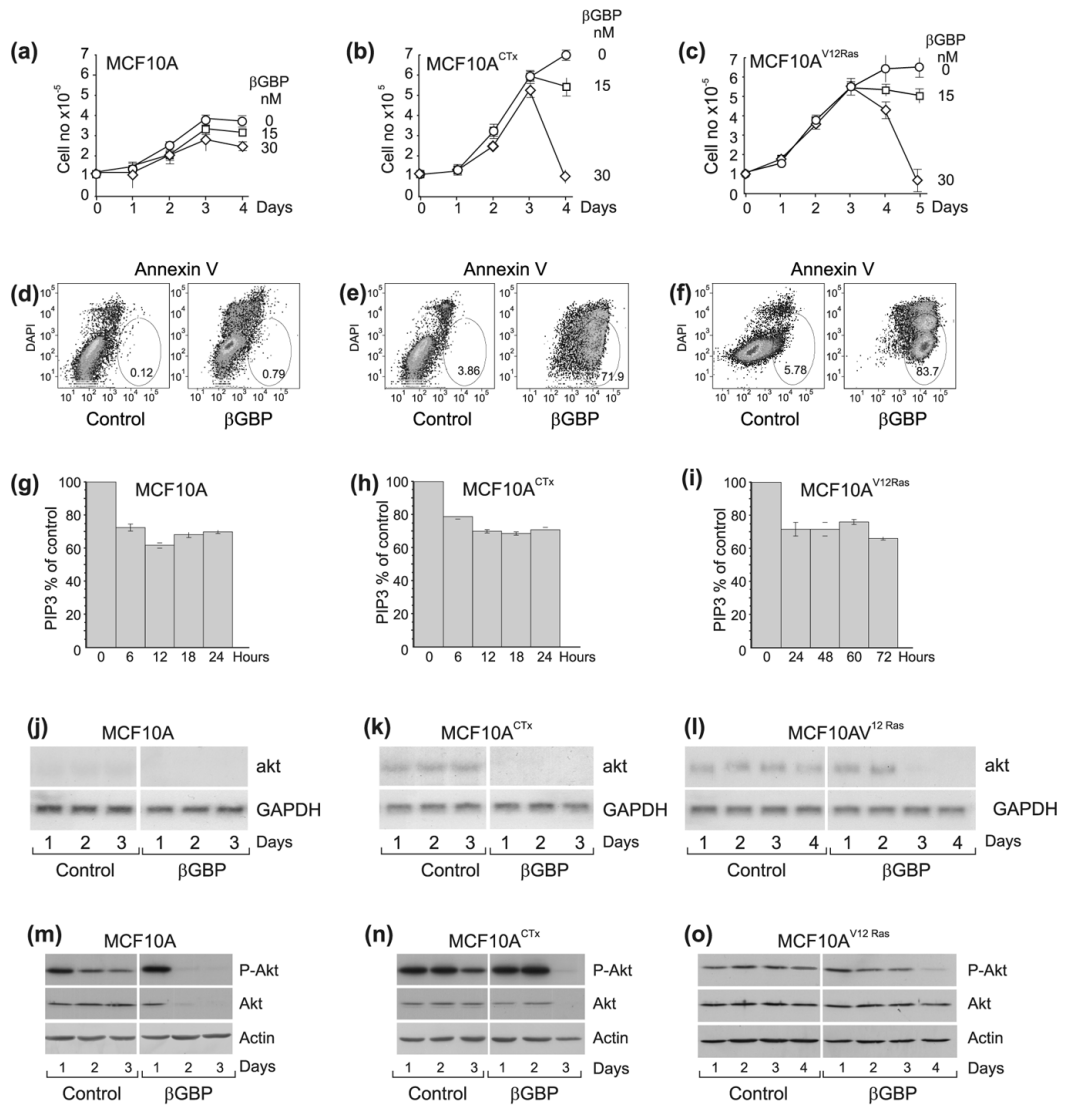
**Effects of cholera toxin (100 ng/ml) and RasV12 on MCF10A breast ductal cells and response to treatment with human recombinant β galactoside binding protein (Hu-r-βGBP)**. **(a-c) **Cell proliferation and dose response to Hu-r-βGBP. Values in growth curves are means from triplicate cultures ± standard error of the mean (SEM). **(d-f) **Apoptotic response to treatment with 30 nM Hu-r-βGBP. Apoptosis assessed by annexin V staining at day 3 after seeding. Apoptotic cells are in circled areas. Values are percentages of cells in apoptosis. Left panels: controls, right panels: cells treated with Hu-r-βGBP. **(g-i) **Inhibition of Class IA phosphoinositide 3-kinase (PI3K) by 30 nM Hu-r-βGBP assessed as described in Figure 1. Values are means from triplicate readings ± SEM. **(j-l) **akt mRNA and glyceraldehyde 3-phosphate dehydrogenase (GAPDH) mRNA levels. Left panels: controls, right panels: cells treated with 30 nM Hu-r-βGBP. **(m-o) **Phosphorylated (Ser473) Akt and total Akt protein. Left panels: controls, right panels: cells treated with 30 nM Hu-r-βGBP. **(a-o) **Hu-r-βGBP added at 3 h after seeding. The data are representative of three separate experiments. Cholera toxin was used to enhance mitogenic signalling via extracellular signal-regulated kinase (ERK) upregulation [35]. Constitutively expressed RasV12 was an endogenous source of enhanced mitogenic signalling [36].

We found that in the naïve MCF10A ductal cells where no extra mitogenic pressure was enforced, treatment with βGBP did not lead to apoptosis (Figure 2a, d). By contrast, when cell proliferation was boosted by cholera toxin or by V12Ras the response to βGBP was characterised by abrupt apoptotic death after 2–3 replication cycles (Figure 2b, c and 2e, f), mimicking the response of the BT474 and SKBR3 cells. Examination of the effect of βGBP on PI3K showed that, as in Figure 1, βGBP had brought down and maintained PI3K activity below basal levels in all cells (Figure 2g–i), but with a delay from 6 to 24 h where the cells were driven by the strong mitogenic signalling imposed by V12 Ras where the apoptotic process was more gradual.

Figure 2 also shows that there was correlation between mitogenic pressure and akt gene expression. Endogenous akt mRNA levels which were barely detectable in the naïve MCF10A cells not subjected to extra mitogenic pressure (Figure 2j, left panel), became clearly expressed where the mitogenic input had been raised, whether by cholera toxin or by V12 Ras (Figure 2k, i, left panels). Significantly, as in Figure 1, inhibition of PI3K activity was followed by loss of akt mRNA (Figure 2j–l) and loss of phosphorylated Akt and Akt protein (Figure 2m–o), but only followed by apoptosis where the akt mRNA levels had been enhanced, a state which, conceivably, conditions cells to vulnerability when exposed to the βGBP cytokine.

**Figure 3:**
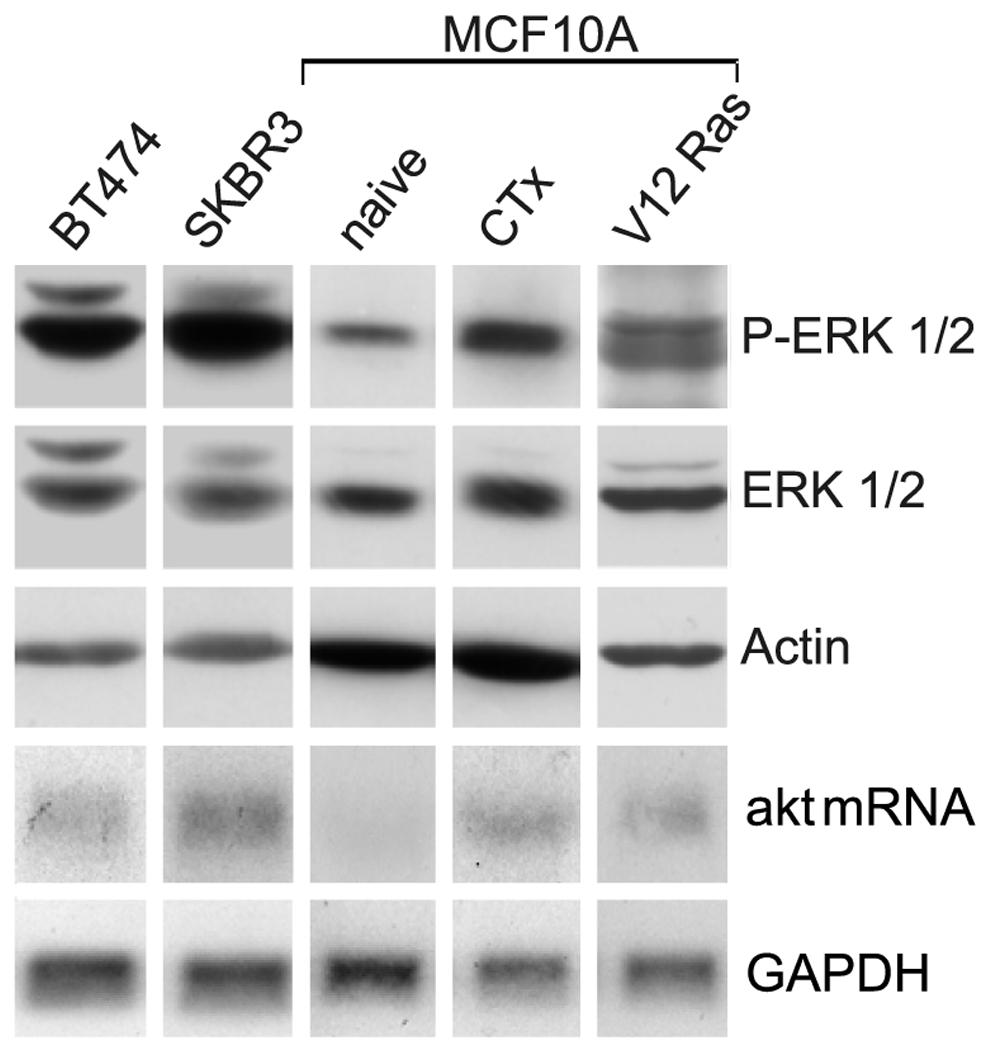
**Extracellular signal-regulated kinase (ERK) levels and Akt mRNA levels as expression of endogenous and enforced mitogenic input in BT474, SKBR3, naïve MCF10A, MCF10A^CTx ^and MCF10A^V12Ras ^cells**. Phosphorylated ERK, total ERK and Akt mRNA assessed in parallel at 48 h or 72 h (SKBR3) during mitogenic expansion. The data are from time scale assessments using equal amounts of protein and equal amounts of RNA and equal exposure times in separate gels due to space limitations when electrophoresing five different cell lines. Repetitions of the experiments gave identical results.

The indication from the above data and that shown in Figure 1 that strong mitogenic input, whether constitutive or induced, is coupled to elevated survival signalling is underscored by the evidence shown in Figure 3, where levels of phosphorylated ERK and levels of akt mRNA (assessed in parallel) correlate. It is of interest within the ERK/akt gene context that our observations bring to attention a putative new aspect in transcriptional control, which extends the role of ERK from the activation of cell cycle promoting genes [37] to the activation of the akt gene, which promotes survival. Attempts to mechanistically validate an ERK/akt mRNA link using MEK-ERK1/2 inhibitors were hampered by poor inhibition or by toxicity not compatible with cell survival.

Notably, we found no evidence that raising active ERK levels, whether by V12Ras or by cholera toxin, had any effect on PI3K activity.

### Cancer phenotype and cell vulnerability

**Figure 4:**
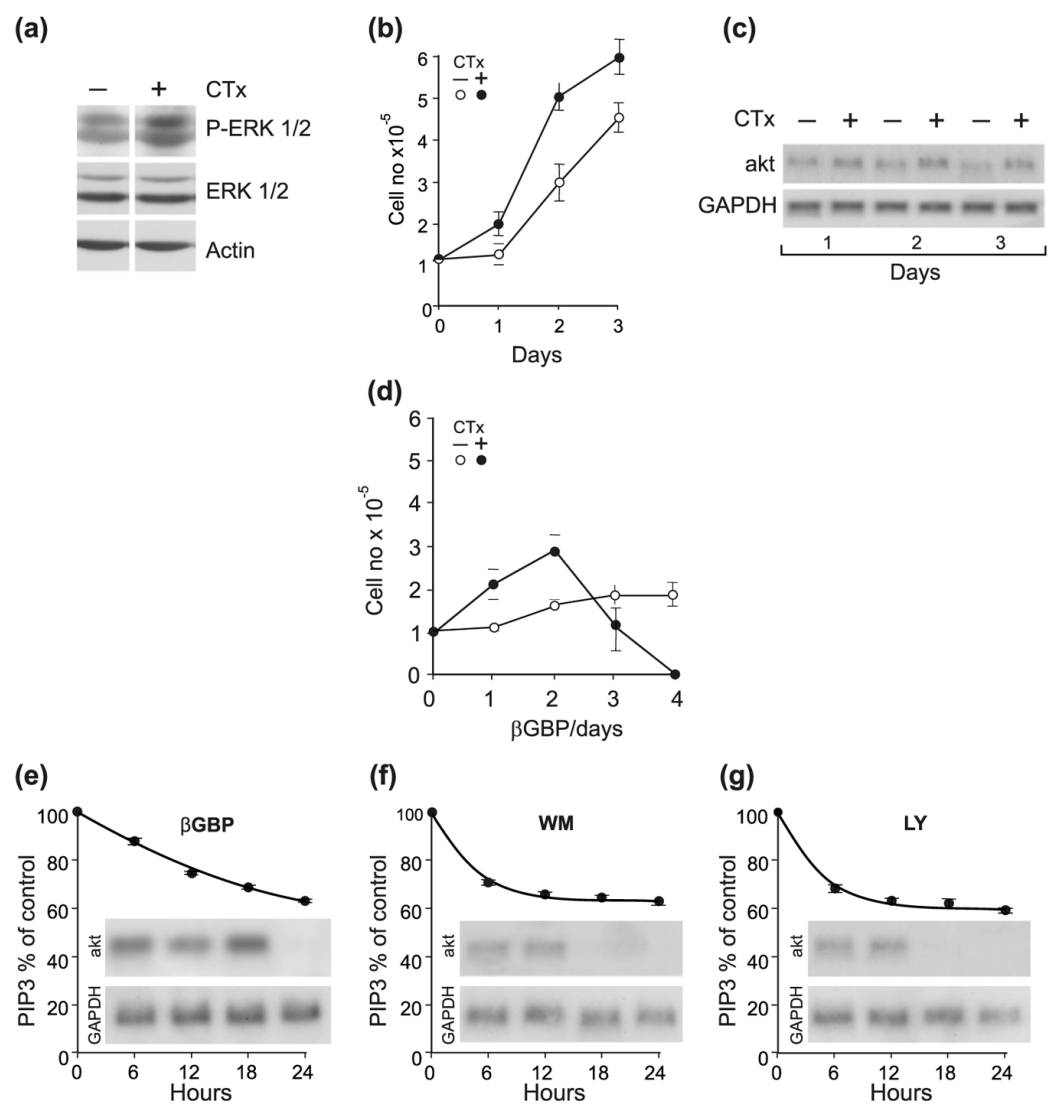
**Response of MCF-7 breast cancer cells to cholera toxin and to cholera toxin and human recombinant β galactoside binding protein (Hu-r-βGBP), and effect of Hu-r-βGBP and phosphoinositide 3-kinase (PI3K) inhibitors on akt gene expression**. **(a-c) **Extracellular signal-regulated kinase (ERK) levels, growth curves and Akt mRNA levels in naïve cells and in cells treated with 100 ng/ml cholera toxin added at the time of seeding. **(a) **Phosphorylated ERK and total ERK assessed at 48 h during mitogenic expansion. **(b) **Naïve cells (open circles); cells treated with 100 ng/ml cholera toxin added at the time of seeding (closed circles). Values are means of triplicate cultures ± standard error of the mean (SEM). **(c) **akt mRNA and glyceraldehyde 3-phosphate dehydrogenase (GAPDH) mRNA levels. **(d) **Growth curves in response to treatment with Hu-r-βGBP in naïve cells (open circles) and in cells treated with 100 ng/ml cholera toxin (closed circles) added at time of seeding. Hu-r-βGBP (20 nM) was added 3 h after seeding. Values are means from triplicate cultures ± SEM. **(e-g) **Effect on downregulation of PI3K activity and akt mRNA in MCF-7^CTx ^cells treated with 20 nM Hu-r-βGBP, 10 nM wortmannin or 20 nM LY294002 added at 3 h after seeding. Phosphatidylinositol (3,4,5)-trisphosphate (PIP3) values are means of triplicate readings ± SEM. The data are representative of three separate experiments.

The evidence that in the MCF10A ductal cells a shift in phenotypic behaviour as a consequence of enforced mitogenic pressure changed the cellular response to βGBP to mimic that of the SKBR3 and BT474 cancer cells, raises the question of whether a shift from a non-aggressive to an aggressive cancer phenotype, as indicated by their *in vitro *behaviour, would increase vulnerability to βGBP. To relate mitogenic input to response to βGBP we examined non-invasive MCF-7 breast cancer cells, which have low levels of ErbB2 [38], in their naïve state and when treated with cholera toxin (MCF-7^CTx^). We found that cholera toxin raised active ERK levels, accelerated cell proliferation and accentuated akt gene expression (Figure 4a–c), thus changing the phenotypic aspect of the cells. Examination of cell response to βGBP showed that while, as reported previously (38,39), in the naïve MCF-7 cells cell replication was inhibited by βGBP (Figure 4d, open circles), the MCF-7^CTx ^cells resisted the growth inhibitory effect of βGBP to succumb, after 1–2 division cycles, to sudden death (Figure 4d, closed circles), again mimicking the response of the BT474 and SKBR3 cancer cells.

Next, we investigated whether PI3K was again a primary responder to the action of βGBP and whether negation of akt gene expression would be the consequence. To secure maximum expression of akt mRNA we used MCF-7^CTx ^cells and carried out time scale experiments using βGBP in parallel with wortmannin [39] and LY294002 [40], both pharmacological inhibitors of the p110 catalytic subunit of PI3K, added at concentrations which would produce an effect similar to that of βGBP, and assessed PI3K activity and akt mRNA levels. Figure 4e–g shows that βGBP lowered PI3K activity to a similar extent as the two inhibitors, but with a more gradual kinetic, in line with the action of a physiological effector molecule, and that akt gene expression was negated when PI3K activity had similarly descended by an approximately 35% quantum below basal levels, in all three instances. This evidence indicates that PI3K activity is a necessary requirement for akt gene expression, and that basal or close to basal endogenous levels are sufficient.

The similarity of the effect exerted by βGBP with that of wortmannin and LY294002 in regard of both inhibitory pattern and the time required for the inhibitory action to come into effect indicates that, as reported previously [32], treatment with βGBP may result in conformational changes which would reduce the functional ability of the catalytic site of the p110 subunit of PI3K [12,13].

## Discussion

The importance of PI3K in the fundamental processes that lead to tumourigenesis has prompted the development of small membrane permeable molecules aimed at targeting components of the PI3K pathway for therapeutic intervention against cancer. The present study suggests that this aim can be achieved using the βGBP cytokine, a natural inhibitor of PI3K [32] whose physiological nature carries no chemotherapeutic disadvantages.

Secreted by CD4^+ ^and CD8^+ ^-activated T cells [31] and by somatic cells [30], endogenous βGBP controls cell cycle entry and S/G2 traverse [30]. In its recombinant form βGBP binds with high affinity (*K*d 1.5 × 10^-10 ^M) to approximately 5 × 10^4 ^receptors/cell, and at a concentration range of 1 to 20 nM βGBP induces inhibition of cell proliferation via S/G2 cell cycle arrest that, while reversible in normal cells, can lead cancer cells to death through routes that, via downregulation of PI3K activity and suppression of Ras-ERK signalling [32], result in cyclin kinase downregulation, deregulated E2F1 transactivation and apoptosis [41]. Cancer cells which respond to βGBP according to this pattern are non-invasive, non-aggressive cells with low levels of ErbB2. They are typified by MCF-7 breast cancer cells and by p53 defective Ramos lymphoma cells [41].

We now report that in breast cancer cells where ErbB2 is overexpressed, βGBP was unable to affect cell proliferation, but, while unable to quench redundant mitogenic signalling and inhibit cell proliferation, by downregulating PI3K activity and suppressing akt gene expression, βGBP had strong therapeutic efficacy that resulted in massive apoptotic death.

The relationship between mitogenic input and akt gene expression and between akt mRNA levels and induction of apoptosis by βGBP as a consequence of downregulation of PI3K activity was validated both in ductal cells and in non-invasive MCF-7 cells where mitogenic signalling was experimentally raised. In the MCF10A ductal cells, once phosphorylated ERK and akt mRNA were boosted by upregulated mitogenic input, and their normal-like behaviour changed to mimic that of the BT474 and SKBR3 cancer cells, loss of akt mRNA resulted in an intensity of apoptotic death similar to that of the BT474 and SKBR3 cells where ErbB2 is overexpressed. In a similar fashion, the MCF-7^CTx ^cells where ERK and akt mRNA had been experimentally upregulated, after overriding the growth inhibitory effect of βGBP, succumbed to total death. This result poses the question of whether, where a shift into malignancy enhances aggressiveness, the use of βGBP might conceivably be a potentially successful alternative to the use of means directed at quenching constitutively active sources of mitogenic signalling.

We have previously reported that luminal breast cells from cosmetic reduction mammoplasties in short-term culture arrested by βGBP suffer no harm and resume growth. Additionally, we have reported that βGBP has no harmful effect on expanding T cells from healthy subjects nor, importantly, on progenitor cells from bone marrow donors [41-43]. In this study, we find that the naïve MCF10A mammary ductal cells suffered little damage when exposed to βGBP indicating that loss of survival signalling is not harmful in the absence of abnormal mitogenic pressure, thus offering one conceivable explanation for the absence of harmful effect by βGBP in the ex vivo normal cells previously studied [41-43]. It is also of interest that when mitogenic input was raised in the ductal cells, the cells underwent apoptotic death when challenged by βGBP. This allows us to speculate that where an increase of mitogenic signalling is a prime occurrence amongst events that lead to oncogenesis, probably nascent cancer cells could be eliminated in the healthy organism by the T cell-produced endogenous βGBP in a surveillance role [31]. A surveillance role for βGBP cytokine may be regarded as a conceivable means by which the immune system may contribute to controlling malignancy.

Taken together, our results suggest a model where high mitogenic input and enhanced ERK activity fosters cell survival by upregulating akt gene expression, for which PI3K activity is a requirement, and where, by downregulating PI3K activity and negating akt gene function, βGBP interrupts cancer cell reliance on survival signalling.

To our knowledge, we have provided the first evidence indicating that PI3K activity is a requirement for akt gene expression and that by targeting PI3K, βGBP can therapeutically suppress akt gene expression and lead to death in tumour cells where the ErbB2 oncoprotein is overexpressed while causing no significant damage to mammary ductal cells.

## Conclusion

PI3K is a central hub of signalling required for cell proliferation and survival, critical in the evolution of aggressive tumourigenesis. The targeting of PI3K by the βGBP cytokine provides a novel mechanistic insight by which the βGBP molecule can overcome ErbB2 aggressiveness, a cause of poor prognosis. The physiological nature of βGBP and its selective efficacy against cells that overexpress ErbB2 indicates that this molecule has the potential to be successfully tested in clinical trials. The study also offers a mechanistic rationale for the use of βGBP against other aggressive conditions, including xeno and self immune responses.

## Abbreviations

βGBP: β galactoside binding protein; DMEM: Dulbecco's modified Eagle Medium; EGF: epidermal growth factor; ELISA: enzyme-linked immunosorbent assay; ERK: extracellular signal-related kinase; PBS: phosphate-buffered saline; PI3K: phosphoinositide 3-kinase; PIP2: phosphatidylinositol (4,5)-biphosphate; PIP3: phosphatidylinositol (3,4,5)-trisphosphate; TUNEL: terminal deoxynucleotidyl transferase (TdT)-mediated dUTP nick-end labelling.

## Competing interests

The authors declare that they have no competing interests.

## Authors' contributions

LM and VW conceived the study and participated in the study design. VW carried out the work. LM drafted the manuscript. LM and VW read and approved the manuscript.
